# Frequency of sexual interactions and associated factors among long-distance truck drivers operating along the Northern Corridor Highway, Kenya

**DOI:** 10.11604/pamj.2021.40.194.31122

**Published:** 2021-12-01

**Authors:** Cyrus Mutie, Salome Kairu-Wanyoike, Susan Mambo, Reagan Ngoge, John Gachohi

**Affiliations:** 1 School of Public Health, Jomo Kenyatta University of Agriculture and Technology, Juja, Kenya,; 2 Meat Training Institute, Ministry of Agriculture, Livestock, Fisheries and Cooperatives, Athi River, Kenya,; 3 Washington State University, Global Health, Nairobi, Kenya

**Keywords:** Casual sexual partners, regular sexual partners, human immunodeficiency virus, risky sexual behavior, sexually transmitted infections

## Abstract

**Introduction:**

harsh working conditions among long-distance truck drivers (LDTDs) expose them to risky sexual interactions while on transit. As a result, the risky sexual interactions among the LDTDs place them at a high risk of contracting human immunodeficiency virus (HIV) and sexually transmitted infections (STIs). This study sought to assess the sexual interactions and associated factors among the LDTDs in Kenya.

**Methods:**

two hundred ninety-six (296) LDTDs were interviewed using interviewer-administered questionnaires. A systematic sampling technique was adopted. The number of sexual acts reported by the respondents was used to generate an ordered outcome variable (frequency of sexual interactions), in the order of; no sexual acts (zero), one to three sexual acts (1), and four to six sexual acts (2). Association between the predictor variables and the outcome variable was analysed using ordered logistic regression analysis in R statistical software.

**Results:**

the mean age of the study participants was 38.4 years, with the youngest being 24 years and the oldest 57 years. Slightly above half of the participants (52.4%) reported no sexual interactions, while the rest (47.6%) had sexual interactions with either casual or regular sexual partners on the week preceding the survey. Age, the number of weeks spent on a transit journey, and drug use were independently associated with the frequency of sexual interactions among LDTDs involved in the study.

**Conclusion:**

the frequencies of sexual interactions are likely to be higher among the younger LDTDs, those who spent more than one week on transit, and those who use alcohol and khat, hence a high exposure risk to HIV/STIs among them.

## Introduction

Long-distance truck drivers (LDTDs) experience harsh working conditions among them long hours of transit without rest, insecurity and high-cost accommodation services, while on transit along the major highways globally [1]. Due to the harsh working conditions, the LDTDs find themselves physically and psychologically exhausted when they stop along the highway to have rest [2,3]. Physical and mental exhaustion may impair their ability to observe safe sexual practices [3]. As a result, the LDTDs engage in risky sexual interactions with their sexual partners.

Drug and substance use, poor condom use, and the presence of casual sex workers are among the main factors known to aggravate the risky sexual interactions among the LDTDs [4-6]. For instance, excess alcohol use is known to impair sound mental judgment, increasing the chances of indulging in risky sexual interactions [3]. The desire to meet sexual needs due to separation from a spouse or a regular sexual partner can also contribute to risky sexual interactions among the LDTDs [1]. In some instances, the LDTDs may not perceive themselves as vulnerable to human immunodeficiency virus (HIV) and sexually transmitted infections (STI) infections [4,7]. While such LDTDs rampantly indulge in sexual interactions, they don´t observe safe sexual practices like good condom use [8]. Consequently, LDTDs are highly likely to contract HIV and STIs due to risky sexual activities, making them a highly vulnerable population. Eventually, they spread HIV and other STI infections to their spouses, sexual partners, and the general population [9].

Efforts in HIV prevention along the major highways have been prioritised on the female sex workers, leaving out their major sexual clients (the LDTDs). Consequently, little is known about the dynamics of sexual interactions among the LDTDs. This, in turn, derails the collective efforts to combat new HIV and STI infections. This is because the strategies that could work on the LDTDs are not adequately exploited, leaving loopholes for new infections. This was part of a larger study that sought to determine the distribution of sexual networks and utilization of HIV/STI preventive services among the LDTDs operating along the Northern Corridor Highway. Some work from the larger study is already published [10]. This study, therefore, sought to assess the frequencies of sexual interactions while at the same time identifying the associated factors.

This study will inform the Ministry of Health and partner stakeholders on the LDTDs who engage in risky sexual interactions and need to be targeted for HIV/STI interventions. The goal is to combat the transmission of new HIV/STIs among LDTDs, their sexual partners, and the general population. Therefore, reduced rates of HIV/STI infections will improve the general human health well-being, hence more economic productivity in the long-distance trucking industry, both in Kenya and other East Africa member countries.

## Methods

**Study design:** we conducted a cross-sectional study among the long-distance truck drivers who operate along the Northern Corridor Highway and make a stopover at the Mlolongo weighbridge in Machakos, Kenya, in July and August 2020. Besides Mlolongo town being a major stopover for weighbridge bureaucracies, it´s also a residential hub for many workers from Nairobi, witnessing a high volume of entertainment activities during weekends hence a suitable stopover for LDTDs [11]. It is also home to robust commercial sex offered by female sex workers, hence a suitable choice for this study.

**Sample size determination and sampling technique:** a sample size of 296 was derived using the Cochran formula [12]:


$$n_{0}=\frac{z^{2}pq}{e^{2}}$$

This formula is used in calculating ideal sample sizes in situations where the study population is large like the case of LDTDs. In this case: z is the number that represents the standard deviations above or below the mean population; a score derived from a z-test which is 1.96; p is the HIV prevalence of LDTDs 26% (0.26) used as the proxy attribute present among the LDTDs operating along the Northern Corridor Highway (0.26) [13]; q is (1 - p), which is (1-0.26) =0.74; e is the desired level of precision (the margin of error), 0.05.


$$n_{0}=\frac{1.96^{2}*0.26*.74}{0.05^{2}}=2.956*100=296$$

A systematic sampling technique was adopted using a daily sampling size of 21 and a sampling interval of 20. A daily sample size of 21 was attained by dividing the study sample size of 296 by the estimated 14 days of data collection. The sampling interval was calculated by dividing the estimated daily number of LDTDs (450) who pass through each of the two weighbridge sections by the daily sample size of 21.

**Data collection:** an interviewer-administered structured questionnaire was used for data collection. The LDTDs were approached towards the exit point of the weighbridge for willingness to participate in the study. Those who agreed to participate were taken through informed consent before the start of the interview. The majority of the LDTDs were willing to be interviewed inside their trucks which they believed offered enough privacy. Those who agreed to participate in the study listed their sexual interaction experiences (history of sexual interactions while on transit, number of sexual acts, type of sexual partners; whether casual or regular) in the week before the day of data collection.

**Data management and analysis:** data were cleaned and later exported to the R statistical software for analysis [14]. Descriptive statistics for socio-demographic and socio-economic characteristics and frequency of sexual interactions were generated. The number of sexual acts reported by the respondents was used to generate an ordered outcome variable, in the order of; no sexual acts (zero), one to three sexual acts (1), and four to six sexual acts (2). Association between the predictor variables and the outcome variable (frequency of sexual interactions) was analysed using ordered logistic regression analysis in R statistical software [14]. All results were presented in tables.

**Ethical consideration:** the ethical review committee of the University of Eastern Africa Baraton approved the study, UEAB/REC/10/02/2020. The study was licensed by the National Commission for Science, Technology and Innovation, Kenya NACOSTI/P/20/4107. At the start of every interview, the study participants were taken through informed consent, after which they would sign the consent form. Confidentiality was ensured by maintaining the anonymity of all LDTDs throughout the data collection, analysis and archiving.

## Results

**Socio-demographic characteristics of the study participants:** the mean age of the LDTDs was 38.4 years, a median of 39.0 years, with the youngest being 24 years and the oldest 57 years. The majority (78.7%) of the LDTDs were of Kenyan citizenship, while the least (2.0%) were from South Sudan. In terms of education, 65.9% of the LDTDs had attained secondary school education, with only 2.0% having attained college-level education. Most of the LDTDs (80.4%) reported that they were married, while the minority (1.4%) had been divorced. In terms of religion, 78.0% of the LDTDs were Christians, while the rest were Muslims (Table 1).

**Table 1:** socio-demographic and socio-economic characteristics of study participants

Variable	Category	Frequency (n=296)	Percentage (%)	95% CI
**Citizenship**	Kenya	233	78.7	73.5, 83.2
	Uganda	36	12.2	8.8, 16.6
	Tanzania	13	4.4	2.5, 7.6
	Rwanda	8	2.7	1.3, 5.5
	South Sudan	6	2.0	0.8, 4.6
**Education**	Primary	72	24.3	19.6, 29.7
	Secondary	195	65.9	60.1, 71.2
	Vocational	23	7.8	5.1, 11.6
	College	6	2.0	0.8, 4.6
**Marital Status**	Single	31	10.5	7.3, 14.7
	Married	238	80.4	75.3, 84.7
	Divorced	4	1.4	0.4, 3.7
	Cohabiting	23	7.8	5.1, 11.6
**Religion**	Christian	231	78.0	72.8, 82.5
	Muslim	65	22.0	17.5, 27.2
**Years working as a truck driver**	Less than one year	5	1.7	0.6, 4.1
	One to three years	45	15.2	11.4, 19.9
	Four to five years	34	11.5	8.2, 15.8
	Six to ten years	66	22.3	17.8, 27.6
	Above ten years	146	49.3	43.5, 55.2
**Weeks away on a transit journey**	Less than seven days	116	39.2	33.6, 45.0
	One week	91	30.7	25.6, 36.4
	Two weeks	83	28.0	23.1, 33.6
	Three weeks	6	2.0	0.8, 4.6
**Income level**	<500 USDs (<50000K.Shs)	285	96.3	93.3, 98.0
	≥500 USDs (≥50000K.Shs)	11	3.7	2.0, 6.7

**Socio-economic characteristics of the study participants:** approximately 49.3% of the LDTDs had work experience of more than ten years, with only 1.7% having worked for less than one year. In terms of weeks spent on a single transit journey, 39.2% of the LDTDs spend less than one week, with only 2.0% spending three weeks. The majority (96.3%) of the LDTDs earned a monthly salary of less than 50,000 Kenya shillings, while the rest earned above 50,000 Kenya shillings monthly (Table 1).

**History and frequency of sexual interactions among study participants:** almost half of the LDTDs (47.6%) had sexual interactions on the week preceding the day of data collection, while the rest did not have sexual interactions. Of those who had sexual interactions, 74.5% had sexual interactions with casual partners and the rest with regular partners. A total of 213 sexual partners had been involved in sexual interactions with the LDTDs. Out of the 213 sexual partners, most of them (82.6%) were casual sexual partners. A total of 428 sexual acts, with an average of 3 sexual acts per sexual interaction, ranging from one to six sexual acts, were reported between the LDTDs and the sexual partners during their sexual interactions (Table 2).

**Table 2:** frequency of sexual interactions among LDTDs along the Northern Corridor Highway, Kenya, 2020

Variable	Frequency (n)	Percentage (%)	95% CI
**History of sexual interaction**	**n=296**		
Yes	141	47.6	41.7, 53.5
No	155	52.4	46.5, 58.2
**LDTDs who reported sexual interaction**	**n=141**		
Had sexual interaction with casual partners	105	74.5	63.3, 81.6
Had sexual interaction with regular partners	36	25.5	18.7, 36.7
**Sexual partners interacted with**	**n=213**		
Casual sexual partners	176	82.6	76.7, 87.3
Regular sexual partners	37	17.4	12.7, 23.3
**Predominant drug use during sexual interactions**	**n=141**		
No drug use	57	40.4	32.3, 49.0
Alcohol	44	31.2	23.8, 39.6
Khat	38	26.0	20.0, 35.2
Marijuana	2	1.4	0.2, 5.6

**Drug and substance use among participants:** out of the 141 LDTDs who had sexual interactions, 59.6% reported drug or substance use during or before sexual interaction. Of the LDTDs who reported substance use during sexual interactions, 31.2% of them had predominantly taken alcohol (Table 2).

**Factors associated with frequency of sexual interactions:** a univariable analysis using the ordered logistic regression was performed to determine the factors associated with the frequency of sexual interactions among LDTDs. The proportional odds ratio (OR) was used to estimate the strength of association between the independent and dependent variables. The level of significance was set at p<0.05, but only the variables with a p-value less than 0.1 at the univariable ordered logistic regression level would enter the multivariable ordered logistic regression analyses. The following variables depicted significance at univariable analyses; age, religion, education, marital status, years working, weeks spent on a transit journey, income level, and predominant drug use (Table 3).

**Table 3:** univariable ordered logistic regression model of factors associated with frequency of sexual interactions among LDTDs, along the Northern Corridor Highway, Kenya, 2020

Variable	Category	OR	95%CI of OR	P-values
**Age**		0.90	0.87, 0.93	**<0.001**
**Religion**	Christian	Ref.		
	Muslim	0.42	0.24,0.74	**0.003**
**Citizenship**	Kenyan	Ref.		
	Non-Kenyans	0.97	0.56,1.66	0.899
**Education**	Primary	Ref.		
	Secondary	1.75	1.03,3.03	**0.004**
	Vocational and collage	2.11	0.94,4.76	**0.007**
**Marital status**	Single or divorced	Ref.		
	Married	0.32	0.17, 0.62	**<0.001**
	Cohabiting	1.34	0.50,3.63	**<0.001**
**Years working**	≤3 years	Ref.		
	4 to 10 years	0.56	0.30,1.07	**<0.001**
	>10 years	0.18	0.09,0.34	**<0.001**
**Weeks away**	<1week	Ref.		
	1 week	1.96	1.13,3.44	**0.002**
	≥2 week	4.23	2.46,7.38	**<0.001**
**Income**	Low income	Ref.		
	Middle lower income	0.10	0.01, 0.54	**0.003**
**Predominant drug use**	No drug	Ref		
	Alcohol	16.4	8.27, 33.8	**<0.001**
	Khat	11.7	5.91, 23.8	**<0.001**
	Marijuana	7.0	0.65, 77.6	**0.010**

Odds ratio (OR), 95% confidence intervals (95% CI), P-values (P<0.05)

With multivariable ordered logistic regression age, the number of weeks spent on a transit journey, and drug use were found to be independently associated with the frequency of sexual interactions among long-distance truck drivers involved in our study, as shown in Table 4. For every one-year increase in age, the odds of reporting 4-6 or 1-3 sexual acts compared to no sexual acts decreased by 9%. For drivers who spent one week on a transit journey, the odds of having at least one sexual act (combining 1-3, and 4-6 sexual acts) versus zero sexual acts was 1.89 times that of drivers who spent less than one week, holding constant all other factors. For drivers who spent at least two weeks on a transit journey, the odds of having at least 1 sexual act (combining 1-3, and 4-6 sexual acts) versus zero sexual acts was 4.16 times that of drivers who spent less than one week on a transit journey, holding constant all other factors. For drivers predominantly using alcohol, the odds of having at least one sexual act (combining 1-3, and 4-6 sexual acts) was 10.9 times that of drivers who used no drug, holding constant all other factors. For drivers predominantly using Khat, the odds of having at least one sexual act (combining 1-3, and 4-6 sexual acts) was 7.05 times that of drivers who used no drug, holding constant all other factors (Table 4).

**Table 4:** multivariable ordered logistic regression model of factors associated with frequency of sexual interactions among LDTDs, along the Northern Corridor Highway, Kenya, 2020

Variable	Category	OR	95%CI of OR	P-values
**Age**		0.91	0.88, 0.95	**<0.001**
**Weeks away**	< 1 week	Ref.		
	1 week	1.89	1.02, 3.53	**0.045**
	Above 2 weeks	4.16	2.25, 7.81	**<0.001**
**Drug use**	No drug	Ref.		
	Alcohol	10.9	5.33, 22.9	**<0.001**
	Khat	7.05	3.46, 14.75	**<0.001**
	Marijuana	3.14	0.27, 37.11	**0.349**

Odds ratio (OR), 95% Confidence intervals (95%CI), P-values (P<0.05)

## Discussion

The study sought to assess the frequencies of sexual interactions and associated factors among the LDTDs who operate along the Northern Corridor Highway segment in Kenya. The mean age of our study participants was 38.6 years, comparable to recent studies conducted in Kenya on LDTDs [3]. A majority, (78.7%) of the LDTDs involved in this study were of Kenyan citizenship, while the rest were from Tanzania, Uganda, Rwanda and South Sudan, suggesting that the trucking industry in Kenya is dominated by drivers of Kenyan origin. Slightly below half, (47.6%) of the LDTDs had been involved in sexual interactions. The LDTDs mostly engaged the casual sex workers in sexual interactions while on transit as compared to the regular sex workers. Young age, spending more than one week on a transit journey and substance use (drinking alcohol and chewing of khat during or before sexual interactions) emerged as the risk factors for a high frequency of sexual interactions among the LDTDs.

Of the LDTDs who reported sexual interactions, 74.5% had sexual interactions with female sexual partners who they were meeting for the first time (casual sexual partners). The rest (25.5%) had sexual interactions with sexual partners they had met before (regular sexual partner). The findings imply that the LDTDs engage casual sexual partners more than regular sexual partners for commercial sex while on transit. As a result, the exposure rates to HIV and STIs are likely to be high among the LDTDs engaging casual sexual partners [15]. Understanding the most engaged group of sexual partners by the LDTDs can inform healthcare stakeholders on priority groups to target with HIV and STI interventions [16]. This approach is not only cost-effective but also saves time and scarce healthcare resources. The study findings are consistent with studies done in South Africa and Bangladesh, which found that casual sex workers are the most engaged by LDTDs along the major highways [1,17]. Contrary to our findings, a study done in Nigeria [18] revealed that 52% of the LDTDs had more sexual interactions with regular sexual partners than with casual sexual partners.

A total of 428 sexual acts were reported from the sexual interactions between the study participants and the female sexual partners. Understanding the number of sexual acts may guide healthcare stakeholders involved in HIV programming on the number of condoms that are needed among the LDTDs and their sexual partners for effective control of HIV and STIs. The study found a lower weekly average of 3 sexual acts among the study participants with their sexual partners, compared to Ferguson and Morris [11], who found a weekly average of 13.6 sexual acts from dairies of FSWs who interacted with LDTDs. The disparity in the results could be explained by the different methods used to collect the data, where this study used self-reporting, while Ferguson and Morris [11] used sex workers' dairies to extract their information on the number of weekly sexual acts.

Regarding drug and substance use during sexual interaction, 31.2% of the LDTDs who reported sexual interactions had taken alcohol as the predominant substance. For effective control of HIV and STIs to be achieved, alcohol use among the LDTDs and their sexual partners should be regulated. Therefore, the transport agencies in the government, healthcare stakeholders, and employers in the trucking industry should work in collaboration to ensure that substance use among the LDTDs is controlled. In India, excessive alcohol use during or before sexual intercourse was found to influence poor condom use [19]. Other studies have identified alcohol use as likely to influence risky sexual behaviours among the LDTDs during sexual interactions [15,18,20,21].

Regarding age, the study established that for every increase in age by one year among the LDTDs, the odds of reporting 1-3 or 4-6 number of sexual acts decreased by 9% compared to zero sexual acts (OR=0.91). The study findings imply that sexual activity decreases with an increase in age among the LDTDs while on transit. Consequently, the younger LDTDs are at a higher risk of exposure to HIV/STIs compared to their older counterparts due to their higher likelihood of engaging in sexual interactions. Thus, HIV/STI preventive services should target young and newly employed LDTDs. Employers in the long-distance trucking industry should also partner with health stakeholders to sensitize newly employed and young LDTDs on the risk of HIV/STI infections that lurks in risky sexual interactions while on transit. Our findings concur with those of Pandey *et al*. [4], which found that LDTDs in India aged 25-34 years were more likely to engage in sexual interactions as compared to their older counterparts.

The study established that spending one week (OR=1.89) and two weeks (OR =4.16) on a transit journey were risk factors for a higher frequency of sexual interactions among the LDTDs. These findings demonstrate that the longer the time LDTDs spend on a transit journey, the higher the number of sexual acts. This outcome highlights the need for employers in the long-distance trucking industry to offer assistant drivers who can relieve the LDTDs who may get exhausted while on long transit. This would shorten the time spent on the transit journey lowering the LDTDs´ exposure to risky sexual interactions and HIV/STIs. Our findings are consistent with a study done in India, which found that LDTDs who spent 15 days and above were more likely to engage in sexual interactions with casual sexual partners compared to those who spent a shorter duration [22].

Similarly, a study done in South Africa [5] found that LDTDs who spent 4 weeks on a transit journey were more likely to engage in sexual interactions and were 1.5 times likely to have HIV infection. Additionally, in India, another study found that spending 10 days and above among LDTDs increased their chances of engaging in paid sexual intercourse [4]. Contrary to our findings, another study done in Iran found no significant association between time spent on a transit journey and the frequency of sexual interactions among LDTDs [21].

On drug and substance use, alcohol use (OR=10.9) and khat (OR=7.05) emerged as risk factors for higher frequencies of sexual interactions among the LDTDs and their sexual partners. These findings indicate that the LDTDs who engage in substance use, specifically alcohol and khat, before or during sexual interactions are more likely to report a higher frequency of sexual interactions than those who do not. The more the frequency of sexual interactions, the higher the exposure risk to HIV and STIs. Therefore, the LDTDs should drink alcohol responsibly while on transit, and where possible, avoid sexual engagements while under alcohol influence. Our findings are consistent with a study done in India that found that LDTDs who used alcohol during or before sexual interactions were more likely to engage in sexual interactions than those who did not [22]. In contrast, a study done in Nigeria found no significant association between substance use and frequency of sexual interactions [18].

**Study limitations:** the use of an interviewer-administered questionnaire in this study may have introduced interviewer bias. The ideal questionnaire recommended in situations of sensitive information (sexual interactions) is the self-administered questionnaire. However, due to the mobile nature of the LDTDs, most of them may not have returned the filled questionnaires, hence a low response rate in the study. The interviewer bias was minimised by training data enumerators on the protocol of handling sensitive responses in the questionnaires while interviewing study participants. Due to the sensitive nature of questions on sexual interactions, responses given by the LDTDs may have suffered social-desirability bias. Social desirability bias occurs when respondents over-report good behaviour and under-report what is not socially welcome in a given social setting. The effect of social desirability bias was minimised through confidentiality and assuring the study participants of the benefits that would come with giving honest responses.

## Conclusion

The key risk factors for a high frequency of sexual activities identified in this study among the LDTDs are; being a LDTD of young age, spending more than one week on a transit journey, and substance use like alcohol drinking and chewing of khat during or before sexual interaction. The study recommends that the ministry of health and other partner stakeholders involved in HIV/STI prevention should improve on targeted interventions among LDTDs who exhibit the above risk factors highlighted. There is a need to enhance awareness and sensitisation on the risk of drug and substance use among the LDTDs. The employers in the trucking industry should also collaborate with the LDTDs in shortening the transit journey to less than one week.

### What is known about this topic

- Female sex workers form the highest proportion of sexual partners who target the long-distance truck drivers for commercial sex along the Northern Corridor Highway.

### What this study adds

- Young age, spending above one week on a transit journey, and substance abuse (alcohol) are independently associated with a high frequency of sexual interactions among the Long-distance truck drivers operating along the Northern Corridor Highway, Kenya.
